# Macrolide resistance in pneumococci—is it relevant?

**DOI:** 10.1186/s41479-016-0010-1

**Published:** 2016-07-07

**Authors:** Allen C. Cheng, Adam W. J. Jenney

**Affiliations:** 1 Infection Prevention and Healthcare Epidemiology Unit, Melbourne, Australia; 2 Microbiology Unit, Alfred Health, Melbourne, Australia; 3 School of Public Health and Preventive Medicine, Melbourne, Australia; 4 Department of Infectious Diseases, Monash University, Melbourne, Australia; 5 College of Medicine, Nursing and Health Sciences Fiji National University, Suva, Fiji

**Keywords:** *Streptococcus pneumoniae*, Antibiotic resistance, Macrolides, Community-acquired pneumonia

## Abstract

Macrolide antibiotics are widely used for a range of indications, including pneumonia. Both high-level and low-level resistance to macrolides is increasing in pneumococci globally. Macrolide resistance in pneumococci is of limited clinical relevance where ß-lactams remain the mainstay of treatment, such as for moderate/severe pneumonia; however, data suggest that macrolides may not be able to be relied on as monotherapy for serious pneumococcal infections.

Macrolide antibiotics, including clarithromycin and azithromycin, remain an important class of antimicrobials for pneumococcal diseases. In Australia, azithromycin is recommended in combination with ceftriaxone as empiric therapy for severe pneumonia, and clarithromycin is a second line therapy for mild/moderate community-acquired pneumonia. United States (US) guidelines are currently being revised, but current recommendations list macrolides as monotherapy for outpatient pneumonia, and macrolides in combination with ß-lactams for more severe pneumonia [1]. Although antibiotics are not routinely recommended for otitis media, there is an exception for high-risk children with otitis media (with or without perforation), in which case azithromycin is listed as one of several therapeutic options [2]. However, the main selection pressure for resistant pneumococci may come from its use in other indications such as for non-pneumococcal respiratory tract infection [3], bronchiectasis and chronic obstructive pulmonary disease (COPD) [4], sexually transmitted diseases and trachoma [5] (in different settings).

The increasing prevalence of macrolide-resistant pneumococci has raised concerns about its place in therapy. There are two major mechanisms mediating resistance to macrolides. The *ermB* gene encodes a methyltransferase that causes ribosomal methylation, resulting in the macrolide-lincosamide-streptogramin B (MLSB) phenotype that reduces susceptibility to macrolides, lincosamide, and streptogramin B. This may be expressed in a constitutive or inducible fashion [6]. *mefA* codes for an antibiotic efflux pump removing the drug from the target site. *ermB* tends to confer high level resistance to macrolides with MICs >64 mg/l, whereas the efflux mechanism results in lower MICs for erythromycin (typically in the 1–16 mg/l range), compared to the European Committee on Antimicrobial Susceptibility Testing (EUCAST) breakpoint for erythromycin (and clarithromycin and azithromycin) of 0.25 mg/l. Other resistance mechanisms also exist, including the *mefE* variant efflux pump carried on the macrolide efflux genetic assembly (mega), mutations in 23S rRNA and also in the L4 and L22 proteins, and the rare *ermA* methyltransferase [7].

There are significant global differences in susceptibility and the mechanisms of resistance. The highest rates of resistance have been reported in East Asia (particularly China, Japan, and South Korea) [8–10] and rapid increases in resistance are occurring in Malaysia [11]. Globally, *ermB* methyltransferase is more common, but the proportion of isolates carrying this gene was higher in several European countries, and less common in North America [12]. Co-existence of both *ermB* and *mefA* is relatively common in some settings. It has been reported at 15 % in South Africa [13], but as high as 38 % in Russia and nearly 50 % in Vietnam [9].

Because of the association between resistance and pneumococcal serotypes, conjugate pneumococcal vaccination has impacted on the epidemiology of resistance. In some places the 7-valent vaccine has been shown to cause a significant and lasting decline in macrolide resistance through reduction in carriage and disease due to serotypes 6B, 9 V, 19 F and 23 F that can carry the *erm* or *mef* genes [14, 15]. While the concerns about replacement with drug-resistant non-vaccine serotypes such as 19A have mostly been addressed by the 13-valent vaccine, replacement with other non-vaccine serotypes and capsular transformation remains a concern [16–19].

Newer macrolides are concentrated intracellularly, and this is thought to result in increased drug delivery to the site of infection, and exposure to high concentrations of drug following phagocytosis, which may overcome low level resistance [20]. However, it has been suggested that high-level resistance may be clinically relevant [21]. A case control study found that 24 % of patients with erythromycin-resistant pneumococcal bacteraemia were taking a macrolide at the time of bacteraemia, compared to none of 136 matched controls with erythromycin-sensitive pneumococcal bacteraemia [22].

The concern about resistance is mitigated by the clinical use of this antibiotic class. There are few indications for macrolide monotherapy for pneumococcal disease. Macrolide monotherapy still has a place in the treatment of community-acquired pneumonia in patients who are allergic to ß-lactams. A meta-analysis compared clinical outcomes in trials involving macrolides, stratified by resistance to azithromycin [23]. Curiously, only 5 of the 13 trials involved community-acquired pneumonia, and the remainder arguably involved patients in whom antibiotics are not indicated, such as chronic bronchitis, acute bacterial sinusitis, and acute otitis media. Although, overall a difference in clinical cure was seen in patients with azithromycin resistance (89.4 % vs. 78.6 %, *p* = 0.003), no differences were evident in patients with pneumonia (94.2 % vs. 92.6 %, *p* = 0.63). Additionally, clinical failure rates across all trials were similar in patients with low-level resistance (77.5 %) compared to high-level resistance (79.2 %).

Another specific indication for macrolides is otitis media in high-risk groups, such as Australian Indigenous children. A clinical trial performed in a setting where resistance was relatively uncommon suggests that clinical outcomes of single dose azithromycin are similar to a 7-day course of amoxicillin, as well as reducing nasal pneumococcal carriage [24]. However, a higher proportion of the children on azithromycin that did carry pneumococci had resistance (10 % vs. 3 %, *p* = 0.001), suggesting that resistance may attenuate this benefit over time.

There are also a number of reasons for the use of macrolides other than their effect on bacteria. There has long been interest in the anti-inflammatory effects of macrolides, and a major indication for its use is in bronchiectasis particularly associated with cystic fibrosis [25, 26]. A 2004 observational study found a large and significance difference in mortality between patients treated with a macrolide-based combination of antibiotics compared with those on monotherapy [27]. Studies have since found a difference in mortality (to a smaller degree) in pneumococcal pneumonia [28, 29]. This difference may possibly be explained by a lower severity of infection associated with macrolide-resistant pneumococci [30].

Clinical trials adding a macrolide to ß-lactams have not definitively demonstrated clinical benefit, but have tested adjunctive macrolides for community-acquired pneumonia generally, rather than pneumococcal pneumonia specifically. One trial found a shorter time to clinical stability in patients with severe pneumonia although the difference in this small trial was not statistically significant [31]. Additionally, there were no differences in other groups or outcomes including length of stay or mortality. A recent cluster randomised trial did not find any differences in mortality or hospital length of stay [32]. The place of adjunctive macrolide therapy in pneumococcal pneumonia remains uncertain.

In summary, macrolide resistance in pneumococci is of limited clinical relevance where ß-lactams remain the mainstay of treatment. However, data suggest that macrolides may not be able to be relied on as monotherapy for serious pneumococcal infections.

## Abbreviations

COPD, chronic obstructive pulmonary disease; MLSB, macrolide-lincosamide-streptogramin B; MIC, minimum inhibitory concentration; EUCAST, European Committee on Antimicrobial Susceptibility Testing
